# Post-trial follow-up methodology in large randomised controlled trials: a systematic review

**DOI:** 10.1186/s13063-018-2653-0

**Published:** 2018-05-30

**Authors:** Rebecca Llewellyn-Bennett, Danielle Edwards, Nia Roberts, Atticus H. Hainsworth, Richard Bulbulia, Louise Bowman

**Affiliations:** 1 MRC Population Health Research Unit, Clinical Trial Service Unit (CTSU), Nuffield Department of Population Health, University of Oxford, Richard Doll Building, Roosevelt Drive, Oxford, OX3 7LF UK; 2 Bodleian Health Care Libraries, University of Oxford, Roosevelt Drive, Oxford, OX3 7LF UK; 3 Molecular and Clinical Sciences Research Institute, St George’s University of London, Cranmer Terrace, London, SW17 0RE UK; 4 Medical Research Council Population Health Research Unit, Nuffield Department of Population Health, University of Oxford, Oxford, UK; 5 Clinical Trial Service Unit and Epidemiological Studies Unit, Nuffield Department of Population Health, University of Oxford, Oxford, UK

**Keywords:** Methodology, Post-trial, Retention, Randomised controlled trial, Cost, Long-term, Follow-up, Effective

## Abstract

**Background:**

Randomised controlled clinical trials typically have a relatively brief in-trial follow-up period which can underestimate safety signals and fail to detect long-term hazards, which may take years to appear. Extended follow-up after the scheduled closure of the trial allows detection of both persistent or enhanced beneficial effects following cessation of study treatment (i.e. a legacy effect) and the emergence of possible adverse effects (e.g. development of cancer).

**Methods:**

A systematic review was conducted following PRISMA guidelines to qualitatively compare post-trial follow-up methods used in large randomised controlled trials. Five bibliographic databases, including Medline and the Cochrane Library, and one trial registry were searched. All large randomised controlled trials (more than 1000 adult participants) published from March 2006 to April 2017 were evaluated. Two reviewers screened and extracted data attaining > 95% concordance of papers checked. Assessment of bias in the trials was evaluated using the Cochrane Risk of Bias tool.

**Results:**

Fifty-seven thousand three hundred and fifty-two papers were identified and 65 trials which had post-trial follow-up (PTFU) were included in the analysis. The majority of trials used more than one type of follow-up. There was no evidence of an association between the retention rates of participants in the PTFU period and the type of follow-up used. Costs of PTFU varied widely with data linkage being the most economical. It was not possible to assess associations between risk of bias during the in-trial period and proportions lost to follow-up during the PTFU period.

**Discussion:**

Data captured during the post-trial follow-up period can add scientific value to a trial. However, there are logistical and financial barriers to overcome. Where available, data linkage via electronic registries and records is a cost-effective method which can provide data on a range of endpoints.

**Systematic review registration:**

Not applicable for PROSPERO registration.

**Electronic supplementary material:**

The online version of this article (10.1186/s13063-018-2653-0) contains supplementary material, which is available to authorized users.

## Background

Randomised controlled trials (RCTs) are considered to be the ‘gold standard‘ for assessing the effects of a treatment. However, these trials usually report results following a relatively brief exposure to the intervention under investigation. Longer-term follow-up of trial participants is important as persistent effects may be detected years later after treatment cessation or even enhanced benefits observed decades later – a so-called ‘legacy effect‘ [1, 2]. Furthermore, delayed hazards may only emerge several years after exposure to certain treatments. Therefore, PTFU may add significant scientific value to the evaluation of many healthcare interventions.

We define post-trial follow-up (PTFU) as extended follow-up which starts after the end of the scheduled period of the trial. Such follow-up, regardless of the primary in-trial outcome, provides important information including safety of the intervention, identification of delayed hazards and long-term beneficial effects.

Retention of participants in PTFU is important since high rates of attrition may introduce bias if reasons for withdrawal are related to the intervention [3]. There are a variety of methods for PTFU, but little research has been done to evaluate which methods for PTFU leads to the best retention rates [4]. Choice of follow-up method is often determined by the funding for the trial and the local availability of relevant data. Telephone calls, postal questionnaires and face-to face interviews are the more traditional approach to follow-up. Web-based approaches and use of routine health records and electronic registries are becoming more popular due to advancing technology and options for accessing the information inexpensively [5, 6].

This systematic review compares methods used in approaches to PTFU and aims to inform the design of PTFU for a wide range of randomised trials. The main objective was to evaluate the retention rates (or levels of attrition) of the participants followed up during PTFU and to compare this to the type of methodology used. A secondary objective was to compare the costs of post-trial methodology as funding is often limited.

## Methods

The methods used in this systematic review have been described in detail previously [7] and follow PRISMA guidelines Additional file 1.

## Eligibility criteria

Briefly, all large (> 1000 adult participants) RCTs which investigated a healthcare intervention (i.e. medicine, surgery or psychiatric in nature) and involved PTFU were included. Only studies published between 2006 and 2017 were included. Alternative medicines (e.g. acupuncture) or holistic interventions including physical therapy were excluded from the review. Large RCTs were only included due to the reduced risk of random error in the outcomes.

PTFU was defined as passive follow-up which had occurred either after the scheduled closure of the trial or after the primary results had been published.

## Search strategy

The search was conducted in five bibliographic databases on 13 April 2017, including Embase (OvidSP) (1 March 1974 to 12 April 2017), Medline (OvidSP) (1946 to present), PubMed, Cochrane Central Register of Controlled Trials (Cochrane Library, Wiley) (issue 3 of 12, March 2017) and Cochrane Methods Register (CENTRAL) (Cochrane Library, Wiley) (issue 3 of 4, July 2017). Searches were then restricted to articles published in English since 2006. Full details of strategies are provided in Additional file 2. In addition, a database search for completed and ongoing studies was conducted at ClinicalTrials.gov (https://clinicaltrials.gov/). Studies which were not yet published ‘grey literature’ were not included in the search strategy.

## Data collection

**Fig. 1:**
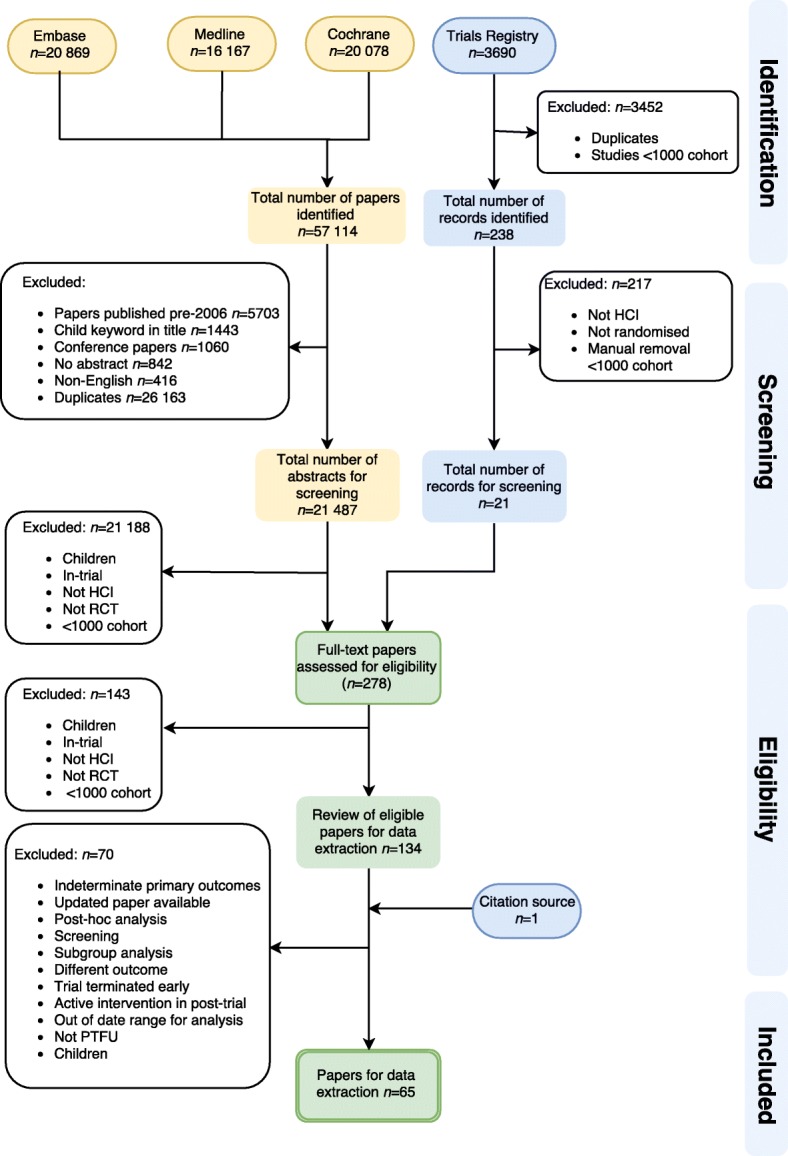
PRISMA flow diagram detailing the process of study selection and data extraction. *HCI* healthcare intervention*, PTFU* post-trial follow-up*, RCT* randomised controlled trial

Papers identified from the ClinicalTrials.gov registry were imported into a MS Excel spreadsheet. Duplicates and studies which had less than 1000 participants were removed using a filter option. The selection of eligible papers followed a concordance strategy between two reviewers (RLB and DE) which ensured that concordance was > 95% (Fig. 1) [7].

Medical interventions were defined as an intervention that was consumed orally, inhaled, or administered by intravenous or intramuscular injections including vaccines. A surgical intervention was defined as any intervention which was invasive (apart from those mentioned above and including blood transfusions). Potential studies were checked for eligibility by two reviewers who initially reviewed abstracts and then proceeded to full paper review in a step-wise process (Fig. 1).

In addition to those described in the protocol, some additional exclusions which were not originally listed were identified during the process of performing the systematic review in keeping with our definition of PTFU. This was required due to the heterogeneity of PTFUs. These include: (1) trials that were stopped before the scheduled closure of the trial; (2) cancer trials which had an open endpoint (e.g. survival as an endpoint with no clear scheduled plan of duration); (3) trials which continued with active intervention in the PTFU period with the primary outcome of safety and (4) trials eligible for inclusion but which did not contribute novel data as they only published additional subgroup or post-hoc analyses. A table of excluded trials is provided in Additional file 3.

Full papers deemed eligible for inclusion in the systematic review were extracted using a standardised Excel spreadsheet. Data was extracted by DE and RLB and concordance was checked. Primary outcome, healthcare intervention and attrition rates were tabulated for each study. Lead trialists were contacted via email to inform them of the systematic review and to clarify information where necessary. The papers included in the review were diverse with a range of interventions and different outcome measures. Due to the high level of clinical heterogeneity a meta-analysis was not possible.

Retention rates were calculated as the proportion of participants who were lost to follow-up compared to the overall total of those who started the PTFU period. Information about the cost of the PTFU was sought from study publications or via personal communication. Two attempts were made to contact the trialist via email and if there was no response or inadequate data, the trial was excluded from the cost analysis.

### Assessment of risk of bias

Risk of bias was assessed for each included RCT on their primary results using the Cochrane Risk of Bias tool. Covdence.org was used to assess the levels of bias (low risk, high risk or unclear risk) in each methodological domain (sequence generation, allocation of sequence concealment, blinding, incomplete outcome data, selective reporting bias and other bias) and decisions checked by one of the senior authors [8]. The data recorded from Covidence.org was imported into Review Manager 5 (RevMan 5) for graphical representation [9].

## Results

From 57,352 papers identified, 65 studies with PTFU were included in the systematic review (Fig. 1). Fifty trials involved medical interventions and 15 involved surgical interventions. There were no eligible psychiatric trials which had (all > 1000 participants). The duration of PTFU ranged from 1 to 20 years, with a median of 4.5 years of follow-up. The number of participants followed during the post-trial period ranged from 575 to 29 862.

Five methods of follow-up were identified: postal correspondence/questionnaire (19%); clinic appointments (35%); telephone interviews (26%); electronic data linkage (52%); and review of paper medical records (26%). In addition, in individual cases, specific follow-up was performed, e.g. endoscopy follow-up only [10]. Electronic data linkage and medical records review were used exclusively together in 11% of papers; either were used in combination with other methods in 74%. Overall, 48% of trials used more than one method to follow-up participants in the post-trial period (Tables 1 and 2). On average, two methods were used for each PTFU follow-up. Where data linkage was used, it was not always feasible to follow up all participants [11]. Some trials experienced difficulty accessing national electronic data in certain countries; for example, stricter regulations are apparent in Canada and for some North American participants (Medicare and Veteran Affairs) where a specific health ID number is required to access national data (Table 3). Trials experienced difficulty in accessing routinely collected health records in 3% of included papers and PTFU was restricted to those countries with robust and accessible centrally held records and registries (e.g. Sweden and Scotland) [12, 13].

**Table 1:** Post-trial follow-up (PTFU) in eligible medical trials. Note retention of participants expressed as % lost to follow-up

1st author, year	Primary outcome for PTFU	RCT name (PTFU name)	No. years PTFU^a^	Intervention	No. randomised in-trial	No. at the start of PTFU	% participants lost in PTFU	Type of PTFU for primary outcome					
								Post/Q	Clinic	Telephone	Data linkage	Paper records	Other
Alan, 2015	Mortality	ProHOSP	6	CAP antibiotics	1359	925	6			Y		Y	
Arbel, 2016	Mortality	BIP	20	Bezafibrate	3090	3090	–				Y		
Arber, 2011	Cancer, safety	PreSAP	2	Celecoxib	1561	1043	12						Y
Avenell, 2012	Mortality	*RECORD*	3	Vitamin D, Calcium	5292	4394	–				Y		
Breitner, 2011	Alzheimer’s disease	ADAPT	2	Naproxen, Celecoxib	2528	2233	1		Y				
Bulbulia, 2011	Mortality and morbidity	HPS	6	Simvastatin	20,536	17519	0	Y			Y		
Cauley, 2013	Hip fractures, cancers, CVE and mortality	WHI	5	Calcium plus vitamin D	36,282	29862	1					Y	
Cherry, 2014	Mortality, cancer	ESPIRIT	12	Oestrogen	1017	1017	–				Y		
Chew, 2013	Progression of age-related macular degeneration	AREDS	5	Antioxidants	4757	3549	–		Y			Y	Y
Chowdhury, 2014	Diabetes mellitus, mortality, MACE	ANBP2	7	ACE inhibitor, Thiazide	6083	5678 (6083 linked to death registry)	–	Y			Y		
Cushman, 2012	MACE, mortality	ALLHAT	13	Amlodipine, lisinopril	32,804	17,722 (CVD), 27,755 (mortality)	–				Y		
Dienstag, 2011	Progression of Hep C	HALT-C	4	Peginterferon	1050	743	–		Y				
Eastell, 2015	Bone mineral density	HORIZON-PFT	3	Zoledronic acid	7765	1223	–		Y				Y
Ebbing, 2010	Mortality	NORVIT, WENBIT	4	B vitamins	6845	6261	0				Y		
Einstein, 2011	Safety, immunogenicity	–	2	HPV vaccine	1106	671	0		Y				–
Erdmann, 2014	Mortality, MI, stroke, MACE, (composite)	PROactive	3	Pioglitazone	5238	3599	9		Y	Y		Y	Y
Ezzedine, 2010	Skin cancer	SU.VI.MAX	5	Antioxidant vitamins	12,741	11054	2	Y				Y	
Flossman, 2007	Colorectal cancer	UK-TIA	20	Aspirin	2449	2249	–				Y	Y	
	Colorectal cancer	BDAT	20	Aspirin	5139	5139	–				Y	Y	
Ford, 2016	Mortality and morbidity	WOSCOPS	20	Pravastatin	6595	5778	–				Y		
Gerstein, 2016	MACE, mortality (composite)	ACCORD (ACCORDIAN)	3	Intensive glucose control	10,251	8601	–		Y	Y			
Gluud, 2008	Mortality	CLARICOR	3	Clarithromycin	4373	4029	1				Y		
Gordon, 2012	Efficacy and safety	REVEAL	2	Adalimumab	1212	575	7		Y				
Grau, 2009	Adenomas	AFPPS	4	Aspirin	1121	1007	14	Y	Y				
Grubb, 2013	Cancer	REDUCE	2	Dutasteride	8231	2751	–		Y	Y			
Hackshaw, 2011	Event-free survival	OVER 50S TRIAL	10	Tamoxifen	3449	3449	–				Y		
Hague, 2016	Mortality, cancer	LIPID	10	Pravastatin	9014	7721	0	Y	Y	Y	Y	Y	
Hayashino, 2009	Diabetes mellitus	PHI1	17	Aspirin	22,071	22,071	–	Y					Y
Hayward, 2015	MACE	VADT	5	Intensive glucose lowering vs standard therapy	1791	1791	22	Y			Y		
Holman, 2008	Macrovascular outcomes	UKPDS	10	Intensive glycaemic control	3867	3277	20	Y	Y		Y		
Hornslien, 2015	Stroke, MI, mortality	SCAST	3	Candesartan	2029	1286	2				Y		
Investigators, 2011	Diabetes mellitus	DREAM (DREAM ON)	2	Rosiglitazone, ramipril	5269	1653	18		Y				
Johnson, 2015	Vaccine efficacy	SPS (LTPS)	4	Vaccine	38,543	6867	6		Y	Y			
Jones, 2015	Cancer, bone fractures	RECORD	4	Rosiglitazone	4447	2546	1			Y	Y	Y	
Kostis, 2011	Mortality	SHEP	13	Chlorthalidone	4736	–	–				Y		
Krane, 2016	MACE, mortality (composite)	4D	8	Atorvastatin	1255	637	3	Y					
Lai, 2014	Mortality, liver cancer	ATBC	16	*α*-tocopherol,β-carotene	29,133	29105	–				Y		
Laterre, 2007	Mortality	ADDRESS	1	Drotrecogin-α	2640	2621	9	Y		Y		Y	
Leslie, 2011	Mortality	ENIGMA	4	Nitrous oxide	2050	2002	17			Y		Y	
Leslie, 2015	MACE, mortality	ENIGMA-II	1	Nitrous oxide	7112	6651	12			Y		Y	
Lewis, 2011	MACE	CAIFOS	5	Calcium	1510	1510	–				Y		
Lloyd, 2013	MACE, cancers, mortality	PROSPER	3	Pravastatin	5804	5188	–				Y		
Menne, 2014	Long-term micro, macrovascular benefit	ROADMAP (ROADMAP OFU)	3	Olmesartan medoxomil	4449	2198	0		Y				
Ogihara, 2011	MACE, cancer, mortality	CASE-J (CASE-J Ex)	3	Candesartan, amlodipine	4728	2232	2		Y				
Radford, 2014	Bone mineral density	Auckland Calcium Study	5	Calcium	1471	1408	17			Y	Y		
Rothwell, 2010	Colorectal cancer	Thrombosis Prev Trial, Swedish Aspirin Low Dose Trial, Dutch TIA Aspirin Trial, UK-Tia Aspirin Trial, British Doctors Aspirin Trial	12, 13, 17, 18, 20	Aspirin	16,488	14033	–		Y		Y	Y	
Tenkanen, 2006	MACE, cancer, mortality	Helsinki Heart Study	10	Gemfibrozil	4081	4081	0	Y			Y		
Wang, 2015	Fracture incidence	NIT	16	Vitamins (14), minerals (12)	3318	3318	1			Y		Y	
Weston, 2011	Persistence of antibodies	106316	3	Vaccine dip, pert, tetanus	2284	1505	–		Y				
Whiteley, 2014	Disability	IST-3	1	Alteplase	3035	2348	2				Y		
Zoungas, 2014	Mortality	ADVANCE (ADVANCE-ON)	6	Perindopril, indapamide	11,140	8494	–		Y	Y			

where ^a^ is number of years (median/mean/max) published in the cited paper, years followed up to the nearest whole number, % participants lost to the nearest whole number,‘–’ no data available or not applicable where mortality records were sought, *CVD* cardiovascular disease, *MACE* major adverse cardiovascular events ± revascularisation, *MI* myocardial infarction. Where 0 participants have been lost to follow-up this has been confirmed either in the cited paper or directly with the corresponding trialist

**Table 2:** Post-trial follow-up (PTFU) in eligible surgical trials. Note, retention of participants is expressed as % lost to follow-up

1st author, year	Primary outcome for PTFU	RCT name (PTFU name)	No. years PTFU ^a^	Intervention	No. participants randomised in trial	No. participants at the start of PTFU	% participants lost in PTFU	Method of PTFU for primary outcome				
								Post/Q	Clinic	Telephone	Data linkage	Paper records
Carson, 2015	Mortality	FOCUS	3	Blood transfusion	2016	2002	–				Y	
Cho 2017	Mortality, MI, stroke, revascularisation	RISPO	4	RIPC, RIPostC	1328	1280	15			Y	Y	Y
Gada, 2013	Safety, efficacy, mortality	SPIRIT III	5	EES, PES	1002	–	–		Y			
Gallagher, 2014	Mortality	RENAL (POST-RENAL)	4	Renal replacement therapy	1508	1464	–				Y	
Halliday, 2010	Mortality, stroke	ACST-1	4	CEA or deferement	3120	3120	–				Y	
Henderson, 2015	Mortality	RITA-3	5	PCI	1810	1810	0				Y	
Hirsch, 2007	Mortality, MACE	ICTUS	4	PCI	1200	1124	3			Y	Y	Y
Hochman, 2011	Mortality, MACE	OAT	3	PCI	2201	1504	–			Y	Y	Y
Investigators, 2007	Mortality	BARI	5	PTCA	1829	1829	4			Y	Y	Y
Milojevic, 2016	Mortality	SYNTAX	5	PCI	1800	847	–		Y	Y		Y
Naunheim, 2006	Mortality	NETT	2	Lung-volume surgery	1218	70%	–	Y			Y	
Patel, 2016	Mortality	EVAR-1	13	EVAR	1252	1252	2		Y		Y	Y
Powell, 2007	Mortality	UKSAT	12	Early AAA repair	1090	1090	0				Y	
Sedlis, 2015	Mortality	COURAGE	6	PCI	2287	1211	–				Y	
Wallentein, 2016	Mortality, MI (composite)	FRISC-II	15	PCI	2457	2421	1				Y	Y

where ^a^ is number of years (median/mean/max) published in the cited paper, years followed up to the nearest whole number, *PCI* percutaneous coronary intervention ± revascularisation, *PTCA* percutaneous transluminal coronary balloon angioplasty, *EES* everolimus-eluting stents, *PES* paclitaxel-eluting stents, *EVAR* endovascular aneurysm repair, *CEA* carotid endarterectomy, *AAA* abdominal aortic aneurysm, *RIPC* remote ischaemic preconditioning, *RIPostC* RIPC with postconditioning, *MI* myocardial infarction, *MACE* major adverse cardiovascular events ± revascularisation, *Postal/Q* postal communication or questionnaire, years followed up to the nearest whole number, % participants lost to the nearest whole number, 70% provided by trialist. Where 0 participants have been lost to follow-up this has been confirmed either in the cited paper or directly with the corresponding trialist

**Table 3:** Registries used for data linkage during post-trial follow-up (PTFU)

Country	Registry	Dataset	Website
USA	United States Renal Data System (USRDS)	Renal	www.usrds.org
	Centres for Medicare and Medicaid Services (CMS ([formerly HCFA))^a^	Non-fatal events	www.cms.gov
	National Death Index Plus Database	Cause- specific mortality	https://www.cdc.gov/nchs/ndi/
	National Death Index and Social Security Administration	All-cause mortality	https://www.cdc.gov/nchs/nvss/deaths.htm
	The Central Veterans Affairs Medical Information files	All-cause morbidity	https://www.va.gov/directory/guide/facility.asp?ID=5380
	The Veterans Affairs Death Files	All-cause mortality	https://www.archives.gov/research/alic/reference/vital-records.html
Canada	Statistics Canada Mortality Database	All-cause mortality	http://www23.statcan.gc.ca/imdb/p2SV.pl?Function=getSurvey&SDDS=3233
England	NHS Digital (formerly HSCIC and Office of National Statistics)	Non-fatal events, all-cause mortality	https://digital.nhs.uk/
Scotland	Information and Statistical Division of the National Health Service for Scotland (Scottish Morbidity Record, General Register Office Death Record)	All-cause morbidity, mortality	http://www.isdscotland.org/
Israel	Ministry of Health from the Israeli Population Registry	All-cause mortality	https://www.health.gov.il/English/Pages/HomePage.aspx
	Israel National Cancer Registry	Cancer	https://www.health.gov.il/English/MinistryUnits/HealthDivision/Icdc/Icr/Pages/default.aspx
Holland	Dutch Central Bureau of Statistics	All-cause mortality	http://www.iamexpat.nl/expat-page/official-issues/organisations/statistics-netherlands-cbs
Norway	Cardiovascular Disease in Norway (CVDNOR) project (for data < 2008)^b^	Cause-specific morbidity	https://cvdnor.b.uib.no/
Finland	Cause-of-Death Register (Statistics Finland)	All-cause mortality	http://tilastokeskus.fi/til/ksyyt/index_en.html
	Population Register Centre ^c^	Demographics	http://vrk.fi/en/frontpage
	Finnish Cancer Registry	Cancer	http://www.cancer.fi/syoparekisteri/en/
Australia	Western Australia Data Linkage System (WADLS)	Non-fatal events, all-cause mortality	http://www.datalinkage-wa.org.au/

^a^ Data only available for those with a valid Medicare or Social Security number (65% of all participants in the ALLHAT long-term follow-up), ^b^Registry linkage in Norway only available from 2008, ^c^ A personal identification number issued to each Finnish resident accesses demographic and medical records

## Retention rates

Unfortunately, retention rates were often poorly reported in the PTFU, limiting the ability to assess the impact of methods used in relation to the proportion lost to follow-up.

All surgical trials investigated mortality as the primary outcome and, where data was available, the proportion of participants lost to follow-up in surgical trials ranged from 0.4 to 15.5%. However, data was not available for 53% of surgical trials. In medical trials, the primary outcome investigated varied more widely, although mortality as an endpoint was common and the proportion of participants lost to follow-up ranged from 0 to 22%. Data on loss to follow-up was not available in 44% of trials. Where mortality was the primary outcome, the number of participants lost to follow-up was not available in 32% of trials due to the use of mortality records where only notifications of deaths were fed back to the trialists.

## Cost

Financial information was available for one third of the included trials. Consequently, it was not possible to provide a direct comparison between cost of PTFU and the different methodologies used due to the small sample size. The cost of PTFU ranged from £6000 to £14,600,000 (Table 4). Cost of PTFU per participant per year showed that IST-3 was the most economical costing £0.21 per participant per year using data linkage/medical records, closely followed by ‘Over 50s’ (£0.41) and RECORD trials (£0.46) which also used data linkage. LTPS was the most expensive PTFU per participant per year (US$531.53) using clinical appointments and telephone follow-up. ROADMAP which also followed up participants by clinic appointment only had a cost of €413.60 per participant per year.

**Table 4:** Comparing post-trial follow-up (PTFU) costs (where disclosed), by different follow-up methodologies

Type of follow-up, name of RCT or PTFU	Number of participants in PTFU	Duration of PTFU*	Incentive for participant follow-up	Cost of PTFU/grant received	Cost per participant per year
Clinical appointment only					
ROADMAP	2198	3.3	Travel reimbursement €20 per visit	€3,000,000	€413.60
Clinical appointment + telephone					
LTPS	6867	4	–	US$14,600,000	US$531.53
Data linkage/medical records only					
RECORD	4394	3	No	£6,000	£0.46
FOCUS	2002	3	No	US$75,000	US$12.49
NORWIT, WENBIT	6261	4	Letters sent to offer withdrawal from PTFU (registry follow-up)	NOK 16,000	NOK 0.64
RENAL	1464	4	No	Undisclosed – original recruiting sites paid for finding and contacting participants	
CLARICOR	4029	3	–	£1,100,000	£91.01
‘Over 50s’	3449	10	no	£14,000	£0.41
RITA-3	1810	5	–	£359,577	£39.73
SCAST	1286	3	no	£7,000	€1.81
CAIFOS	1510	4.5	no	AUD 848,206	AUD 124.83
IST-3	2348	1	no	£500	£0.21
Telephone + data linkage/medical records					
ProHOSP	925	6	no	Negligible. Students conducted telephone follow-up as part of their training	–
OAT	1504	3	no	US$100 administrative start-up, US$50 per call for each follow-up, US$30 per subject for re-consent payment, US$300 per event completing reporting	–
ENIGMA	2002	3.5	no	AUD 53,807	AUD 7.68
ENIGMA-II	6651	1	no	AUD 60,000	AUD 9.02
Postal correspondence + data linkage/medical records					
HPS	17,519	6	–	£250,000	£2.38
ANBP2	6983	6.9	no	AUD 18,000	AUD 0.37
ACST-1	3120	4	–	£120,000	£9.62
VADT	1791	5	US$10 per survey gift card	US$10,00,000	US$111.67
Postal correspondence +telephone + medical records					
ADDRESS	2621	1	no	US$13,10,500	US$500

where ^a^; median/max/range published in the cited paper, *RCT* randomised controlled trial, *PTFU* post-trial follow-up, *NOK* Norwegian Krone, *AUD* Australian dollar; ‘*-’* no data available/ declined by corresponding trialist, ‘~‘ ,estimate; + RCT number as PTFU data not available. Results to 2 decimal places for cost per participant

## Cochrane Risk of Bias

We hypothesised that those RCTs which had poor methodology or ‘high risk of bias’ might subsequently have a PTFU which was poorly organised and, therefore, have low retention rates (or a high proportion lost to PTFU). Of the 65 papers included in the systematic review, seven were excluded from the risk of bias assessment: these were PTFUs which followed-up an amalgamation of data from more than one trial or were part of a systematic review and, therefore, not suitable to be included in the analysis (the risks of bias from individual component trials could not be combined).

**Fig. 2:**
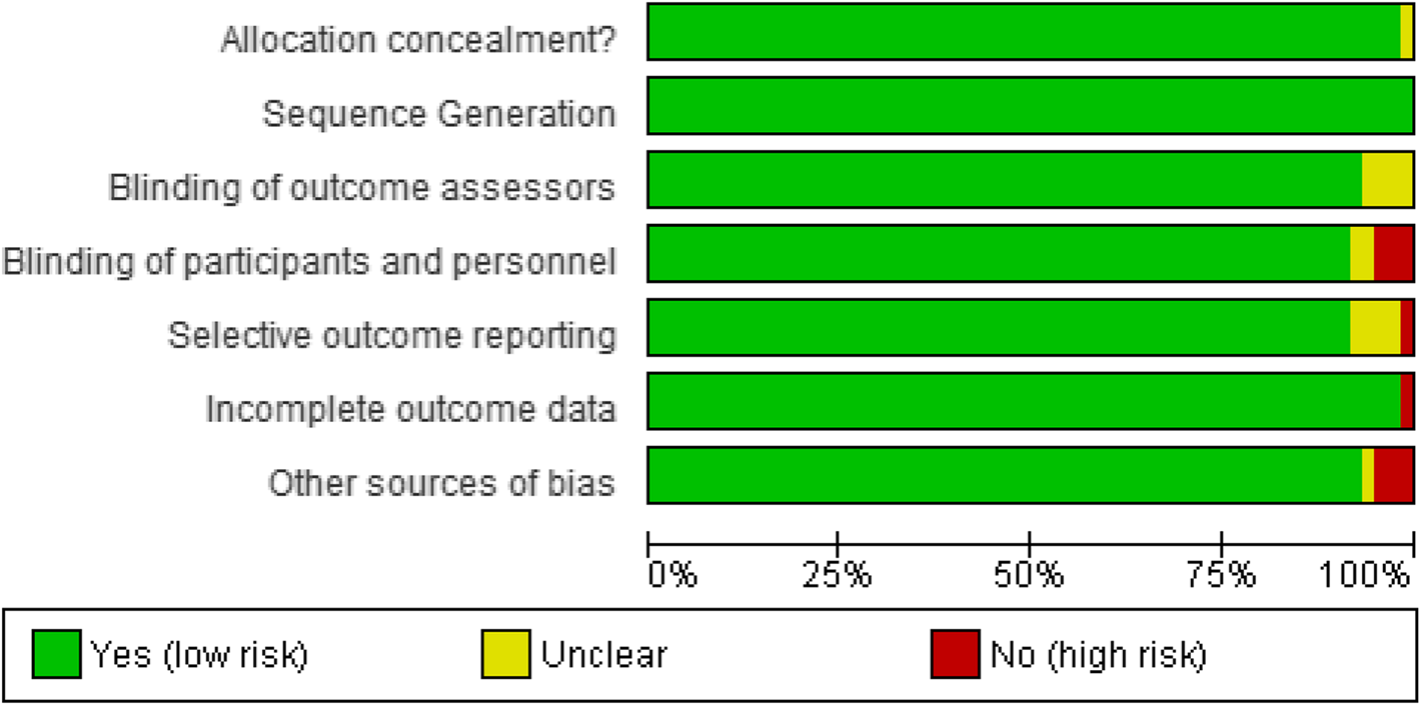
Cochrane Risk of Bias graph. Review authors’ judgements about each risk of bias item presented as percentages across all included studies

Of the 58 trials considered, the risk of bias could not be fully assessed in 11 trials due to lack of information in at least one domain. Low (or unclear) risk of bias in all domains was found in 43 (74%) of those assessed. Only seven trials (12%) had at least one domain which was high risk of bias, of which one had two domains at high risk (Fig. 2). Details of the individual risk of bias domains for each included study are provided in Additional file 4.

Given the small number of trials found to have a high risk of bias in at least one domain and the highly variable retention rates observed during PTFU (Table 5), it is not possible to draw any clear conclusions with respect to possible associations between risk of bias and its potential impact on the proportion of participants that were lost to follow-up in the post-trial period.

**Table 5:** Comparison of randomised controlled trials (RCTs) which had high risk of bias compared to the proportion lost to follow-up during post-trial follow-up (PTFU). A summary of those RCTs with no risk of bias are also detailed

High-risk domain	Number of studies with high-risk domain	Proportion of participants lost to follow-up during PTFU (%)
Blinding participants and personnel	3	3.96–6.16
Incomplete outcome data	2	–
Other sources of bias	3	1.21–11.79
Selective outcome reporting	1	1.2
Low risk of bias in all domains	43 (no high/unclear risk of bias)	0–19.90

## Discussion

This systematic review identified that PTFU methods varied and many trials used overlapping approaches which were more costly than needed. Data was limited on retention rates and so it was difficult to draw any firm conclusions on which method was best for PTFU.

Our main findings suggest that most PTFU published in the last 11 years does not appear to be designed in a cost-effective manner. Cost of PTFU was shown to vary widely and not many trials used incentives to retain participants. Despite only a third of trialists providing complete financial information for PTFU, follow-up by clinical appointment appeared to be the most expensive method, as might be expected given the resource implications. Postal or telephone correspondence in addition to data linkage did not appear to increase the cost per participant per year considerably. However, the effect of inflation over the 11 years included in this systematic review, makes quantitative comparison of cost differences difficult. Given the limited data available we have not attempted to adjust for inflation.

Data linkage or access to medical records is likely to be the most cost-effective method of following participants due to minimal staff required. However, a number of trialists highlighted the limitations of this approach, noting it to be time-consuming and frustrating with increasing regulatory costs and country-specific restrictions. In the UK, the process of accessing data electronically has become more stringent and costly, and markedly different to the processes which were encountered 10 years ago. There is also an issue of the data lagging behind by up to 2 years in some countries, which can impact on the completeness of results for a trial. Despite this, data linkages to national registries and electronic health records have been shown to be a valid and reliable method of PTFU [12–15].

When designing this systematic review, we anticipated that papers published in the early half of the last 11 years would choose more traditional methods of PTFU, e.g. clinic- and telephone-based approaches, and more recent trials may increasingly use data linkage where available. However, this has not been the case. The majority of trials have used a variety of different methods to capture data for the same primary outcome. We were, therefore, unable to compare retention rates by each type of method used. In addition, sparsity of complete data in the review (typically poor reporting of the final number of participants at the end of the follow-up period) limited the ability to assess retention rates achieved with different PTFU methods.

We found limited evidence of high risk of bias in the methodology of the in-trial periods. A likely explanation for this is that the majority of the trials included in this review were well-designed, large RCTs in which results were published in high-impact journals. Furthermore, trials which employ poor methodology or have had negative results are more likely not to engage in PTFU due to lack of funding or interest.

Due to new guidelines (Consolidated Standards of Reporting Trials (CONSORT)) recommending increasing transparency in the reporting of RCTs, a more complete capture of data would be likely for any future study [16]. Research into appropriate methods in PTFU can only occur if there is transparency of the logistical and financial implications including number of participants lost to follow-up.

## Conclusions

Post-trial follow-up of large RCTs can contribute significantly to the scientific value of a trial by determining the longer-term magnitude of the effects of an intervention. PTFU is valuable to ensure that there are no long-term hazards or beneficial effects which have been missed due to the common short in-trial periods for following up participants. However, it is not widely used as shown by the small number of eligible trials which had PTFU from the original search strategy.

Data linkage and the use of registries appear to be the most plausible and economical approach to PTFU. These methods also have the advantage of providing data for a wide range of endpoints. Improvement of electronic reporting and informatics could lead to better reporting and allow this type of method to be widely used.

## Additional files

Additional file 1: PRISMA Checklist. (DOCX 30 kb)

Additional file 2: Search strategies. Key to operators used in Medline/Ovid: where .pt. is publication type, (?) represents any single character, (*) is a group of characters, .mp.is multi-purpose search, /is Medical Subject Headings (MeSH), exp. is explode subject heading, .sh. is subject heading, (““) is phrase search. Comments: all results were downloaded with all fields displayed and in a tab delimited format. This file was then opened in Excel. Duplicates were removed. The spreadsheet sort order was changed to Enrollment A–Z and studies with fewer than 1000 enrollees will be removed. (PDF 396 kb)

Additional file 3: Trials with long-term follow-up excluded from final analysis. *open-label study investigating safety doses of intervention. Extension study of two previous RCTs (Philipp T et al. Clin Ther 2007; 29:563–80). (PDF 147 kb)

Additional file 4: Risk of bias shown in each domain for an individual randomised controlled trial (RCT). Red indicates high risk, yellow indicates unsure and green indicates low risk. (PDF 173 kb)

## Abbreviations

CENTRAL: Cochrane Central Register of Controlled Trials
EMBASE: Excerpta Medica database
PTFU: Post-trial follow-up
RCT: Randomised controlled trial
