# Supplementary material for: Post-trial follow-up methodology in large randomised controlled trials: a systematic review
Source: Trials. 2018 May 30;19:298. doi: 10.1186/s13063-018-2653-0 (PMC5975470; doi:10.1186/s13063-018-2653-0)
Supplement: Supplementary file 1 — PRISMA Checklist. (DOCX 30 kb) [file 13063_2018_2653_MOESM1_ESM.docx]

| **Section/topic** | **#** | **Checklist item** | **Reported on page #** |
| --- | --- | --- | --- |
| **TITLE** | | |  |
| Title | 1 | Post-trial follow-up methodology in large randomised controlled trials: a systematic review | 1 |
| **ABSTRACT** | | |  |
| **Structured summary** | 2 | A systematic review was conducted following PRISMA guidelines to qualitatively compare post-trial follow-up methods used in large randomized controlled trials. Four bibliographic databases, including Medline & Cochrane Library, and 1 trial registry were searched. All large randomized controlled trials (more than 1000 adult participants) published from March 2006 - April 2017 were evaluated. Assessment of bias in the trials was evaluated using the Cochrane Risk of Bias tool. The protocol detailing the methods has already been published.  57 352 papers were identified and 65 trials which had post-trial follow-up (PTFU) were included in the analysis. The majority of trials used more than one type of follow-up. There was no evidence of an association between the retention rates of participants in the PTFU period and the type of follow-up used. Costs of PTFU varied widely with data linkage being the most economical. There was no association between risk of bias in the in-trial period and proportions lost to follow-up during the PTFU period.  Data captured during the post-trial follow-up period can add scientific value to a trial. However, there are logistical and financial barriers to overcome. Where available, data linkage via electronic registries and records is a cost-effective method which can provide data on a range of endpoints. | 2 |
| **INTRODUCTION** | | |  |
| Rationale | 3 | Randomised controlled trials (RCT’s) are the gold standard for investigating an intervention. However RCT’s are costly and usually employ a brief treatment period with limited in-trial follow-up. It is questionable, that an initial response to a treatment can predict a beneficial long-term outcome. Post- trial follow-up can detect persistent treatment effects, safety issues or enhanced benefits that were not apparent during in-trial. However, there is no international consensus on the methodology for post-trial follow-up. | 3 |
| Objectives | 4 | The design of this systematic review is to qualitatively compare post-trial follow-up methodologies used in large randomised controlled trials. Our objectives are to compare retention rates of participants and costs of different methods used in post-trial follow-up. | 3 |
| **METHODS** | | |  |
| Protocol and registration | 5 | Post-trial follow-up methodology in large randomized controlled trials: a systematic review protocol <https://systematicreviewsjournal.biomedcentral.com/articles/10.1186/s13643-016-0393-3> | 3 |
| Eligibility criteria | 6 | All published large randomized controlled trials of >1000 participants which had post-trial follow-up published between 2006- 2016. The title and abstract must be in English.(see table 1) | 4 |
| Information sources | 7 | From April 2006- April 2017. Electronic databases will include Cochrane methodology group register, Cochrane Central Register of Controlled Trials (CENTRAL), Medline, Embase and trials registries (clinical-trials.gov) | 4 |
| Search | 8 | See Appendix 1 for MESH and defined keywords | 4 |
| **Study selection** | 9 | See PRISMA figure 1, further details published in protocol. | 4 |
| Data collection process | 10 | See PRISMA figure 1 and published protocol. | 4 |
| Data items | 11 | Retention rates (or participants lost to follow-up), missing data, costings, length of post-trial follow-up, numbers of participants followed-up compared to in-trial numbers. Additional information provided in published protocol | 4-7 |
| Risk of bias in individual studies | 12 | Cochrane Risk of Bias tool will used at the study level | 7 |
| Summary measures | 13 | \| - No data synthesis planned \| \| --- \| \| - Summary of heterogeneous data will be compared in tables \| \| - Cochrane Risk of Bias will be used. \| | 4-7 |
| Synthesis of results | 14 | - Summary of heterogeneous data will be compared in tables | 5-6 |

Page 1 of 2

| **Section/topic** | **#** | **Checklist item** | **Reported on page #** |
| --- | --- | --- | --- |
| Risk of bias across studies | 15 | Not applicable | - |
| Additional analyses | 16 | Not feasible | - |
| **RESULTS** | | |  |
| Study selection | 17 | See figure 1 | 5-7 |
| Study characteristics | 18 | See table 1 and 2 | 5-7 |
| **Risk of bias within studies** | 19 | See figure 5 and Appendix C | 7 |
| Results of individual studies | 20 | See Appendix C | 7 |
| Synthesis of results | 21 | Table 5, Figure 2, Appendix C | 5-7 |
| Risk of bias across studies | 22 | See figure 5 and Appendix C | 7 |
| Additional analysis | 23 | Not feasible | - |
| **DISCUSSION** | | |  |
| Summary of evidence | 24 | This systematic review identified that post-trial follow-up varied and many trials used overlapping methods and were more costly than needed. Data was limited on retention rates and so it was difficult to draw any firm conclusions on which method was best for post-trial follow-up. | 7-9 |
| Limitations | 25 | See Discussion | 7-9 |
| Conclusions | 26 | See Conclusion. | 9 |
| **FUNDING** | | |  |
| Funding | 27 | Royal College of Surgeons Research Fellowship funding granted to Rebecca Llewellyn-Bennett | 10 |

*From:*  Moher D, Liberati A, Tetzlaff J, Altman DG, The PRISMA Group (2009). Preferred Reporting Items for Systematic Reviews and Meta-Analyses: The PRISMA Statement. PLoS Med 6(7): e1000097. doi:10.1371/journal.pmed1000097

For more information, visit: **www.prisma-statement.org**.

Page 2 of 2
