# Supplementary material for: Post-trial follow-up methodology in large randomised controlled trials: a systematic review
Source: Trials. 2018 May 30;19:298. doi: 10.1186/s13063-018-2653-0 (PMC5975470; doi:10.1186/s13063-018-2653-0)
Supplement: Supplementary file 2 — Search strategies. Key to operators used in Medline/Ovid: where .pt. is publication type, (?) represents any single character, (*) is a group of characters, .mp.is multi-purpose search, /is Medical Subject Headings (MeSH), exp. is explode subject heading, .sh. is subject heading, (““) is phrase search. Comments: all results were downloaded with all fields displayed and in a tab delimited format. This file was then opened in Excel. Duplicates were removed. The spreadsheet sort order was changed to Enrollment A–Z and studies with fewer than 1000 enrollees will be removed. (PDF 396 kb) [file 13063_2018_2653_MOESM2_ESM.pdf]

## Appendix A: Search Strategies

### Medline Search strategy

- Search conducted via OvidSP interface: 1946-present in process & other non-indexed citations

| Step | Search domain | Search terms |
|---|---|---|
| s1 | Randomized controlled trials | randomized controlled trial.pt. or random?ed control* trial*.mp. or random allocation/ |
| 2 | Post- trial | (Post-trial or post trial or attrition or drop out? or dropout? or follow-up or followup? or extension* or trial closure? or long-term or longterm or extended observation* or extended stud*).mp. |
| 3 | Outcomes | (cost effective* or cost benefit* or costs or survivors or hospital admission? or hospitali?ation? or primary outcome* or secondary outcome* or primary endpoint? or secondary endpoint? or composite endpoint? or primary end point? or secondary end point? or composite end point? or outcome measure* or outcome assessment or outcomes research).mp. or treatment outcome/ |
| 4 | Types of methodological follow-up | electronic data processings/ or automatic data processing/ or "surveys and questionnaires"/ or telephone/ or interview/ or data collection methods/ or data collection/ or data linkage/ or data system?.mp. or data reporting/ or epidemiologic method?.mp. or incidence.mp. or mortality.mp. or health episode statistics.mp. or electronic health record?.mp. or electronic patient record?.mp. or computeri?ed record-linkage system?.mp. or national register*.mp. or national database*.mp. or episode of care/ or routine data.mp. or routinely collected data.mp. |
| 5 | | 1 and 2 and 3 or 4 |
| 6 | 5 | exp animals/ not humans.sh. |
| 7 | 6 | limit to (abstracts and English language and yr="2006 - Current") |

*Key to operators used in Medline/Ovid: where .pt is publication type, (?) represents any single character, (*) is a group of characters, .mp.is multi-purpose search, /is Medical Subject Headings (MeSH), exp is explode subject heading, .sh. is subject heading, (“ “) is phrase search.*

## Embase Search Strategy

- Search conducted via OvidSP interface 1st March 1974-12 April 2917

| Step | Search terms |
|---|---|
| 1 | exp medical record/ or interview/ or telephone interview/ or survey?.mp. or questionnaire?.mp. or data system?.mp. or epidemiologic method?.mp. or incidence.tw. or mortality.tw. or cardiovascular mortality/ or cancer mortality/ or *mortality/ or health episode statistics.mp. or electronic health record?.mp. or electronic patient record?.mp. or computeri?ed record-linkage system?.mp. or national registr*.mp. or national database*.mp. or routine data.mp. or routinely collected data.mp. |
| 2 | (cost effective* or cost benefit* or costs).mp. or clinical effectiveness/ or effectiveness.mp. or survivors.mp. or hospital admission?.mp. or hospital episode?.mp. or hospitali?ation?.mp. or primary outcome*.mp. or secondary outcome*.mp. or outcome measure*.mp. or outcome assessment.mp. or outcomes research.mp. or treatment outcome/ or treatment duration/ |
| 3 | randomized controlled trial/ or ((random* or blind* or placebo*).tw. and major clinical study/) |
| 4 | (Post-trial or posttrial or attrition or drop out? or dropout? or follow-up or followup? or extension* or trial closure? or long-term or longterm or extended observation*).mp. |
| 5 | 1 and 2 and 3 and 4 |
| 6 | (exp animals/ or nonhuman/) not human/ |
| 7 | 5 not 6 |
| 8 | limit 7 to (english language and yr="2000 -Current") |
| 9 | (letter or editorial or conference*).pt. |
| 10 | 8 not 9 |

## Cochrane Library Search Strategy

- Search conducted via Cochrane Library via Wiley interface
- Cochrane Central Register for Controlled Trials *(Issue 3 of 12, March 2017)*
- Cochrane Methods Register *(Issue 3 of 4, July 2017)*

| Step | Search terms |
|---|---|
| 1 | MeSH descriptor: [Surveys and Questionnaires] this term only |
| 2 | MeSH descriptor: [Data Collection] this term only |
| 3 | MeSH descriptor: [Interview] explode all trees |
| 4 | MeSH descriptor: [Automatic Data Processing] this term only |
| 5 | data collection or "data processing*" or "data system*" or "data linkage" or "data reporting" or incidence or mortality or "health episode statistics" or "electronic health record*" or "electronic patient record*" or "record linkage system" or "record-linkage system*" or "national registr*" or "national database" or "episode of care" or "routine data" or "routinely collected data":ti,ab,kw (Word variations have been searched) |
| 6 | 1 or 2 or 3 or 4 or 5 |
| 7 | cost effective* or "cost benefit*" or costs or "clinical effectiveness" or survivors or "hospital admission*" or hospitalisation* or hospitalization* or "primary outcome*" or "secondary outcome*" or "outcome measure*" or "outcome assessment" or "outcomes research" or "treatment outcome":ti,ab,kw (Word variations have been searched) |
| #8 | post trial or posttrial or attrition or "drop out*" or dropout* or "follow up" or followup* or extension or "trial closure*" or "long term" or longterm or "extended observation*":ti,ab,kw (Word variations have been searched) |
| #9 | #6 and #7 and #8 |

## Clinical Trials Search Strategy

- http://clinicaltrials.gov

| | Search terms | Limits |
|---|---|---|
| 1 | post-trial OR "post trial" OR posttrial OR attrition OR "drop out" OR dropout OR extension OR extended | Studies With Results<br>Interventional Studies |
| 2 | longterm OR long-term OR "long term" OR follow-up OR "follow up" OR followup | Studies With Results<br>Interventional Studies |
| | **1 or 2** | |

**Comments:**

All results were downloaded with all fields displayed and in a tab delimited format. This file was then opened in ExCel. Duplicates were removed. The spreadsheet sort order was changed to Enrollment A-Z and studies with fewer than 1000 enrolees will be removed.
