# Supplementary material for: Post-trial follow-up methodology in large randomised controlled trials: a systematic review
Source: Trials. 2018 May 30;19:298. doi: 10.1186/s13063-018-2653-0 (PMC5975470; doi:10.1186/s13063-018-2653-0)
Supplement: Supplementary file 3 — Trials with long-term follow-up excluded from final analysis. *open-label study investigating safety doses of intervention. Extension study of two previous RCTs (Philipp T et al. Clin Ther 2007; 29:563–80). (PDF 147 kb) [file 13063_2018_2653_MOESM3_ESM.pdf]

Appendix B: Trials with long-term follow-up excluded from final analysis

Exclusion Criteria	Name of 1st Author	Year of PTFU publication	Name of Trial/Title of paper (ABBREVIATION where possible)
Indeterminate primary outcomes			
	Bosset J	2014	EORTC 22921
	Collaborative Ocular Melanoma Study Group	2006	COMS
	Cuzick J	2014	IBIS-II
	Donker M	2013	EORTC 10853
	Ligibel J	2015	CALGB 9741
	Lundstedt D	2010	SWEBCG91-RT
	Poortmans PM	2015	EORTC 22922/10925
	Regan MM	2011	BIG 1-98
	Sparano J	2015	E1199
	Van Gijn W	2011	TME Trial
Post-hoc analysis of included paper			
	Abedini S	2009	ALERT
	Abedini S	2009	ALERT
	Abedini S	2010	ALERT
	Adlbrecht C	2014	OAT
	Alderman M	2016	ALLHAT
	Alva ML	2015	UKPDS
	Chowdhury E	2015	ANBP2
	Lotan Y	2012	SELECT
	Wilson R	2009	Assessment of survival in a 2-year comparative study of lanthanum carbonate versus standard therapy
Screening randomised controlled trials			
	Oken M	2011	PLCO
	Schroder F	2014	ERSPC
	Swanson S	2015	Norwegian Colorectal Cancer Prevention Trial
	van Leeuwen PJ	2012	ERSPC
Subgroup analysis of included paper			
	Barzilay J	2012	ALLHAT
	Brown L	2008	OAT
	Kappetein AP	2013	SYNTAX
	Kataja-Tuomola MK	2010	ATBC
	Mack MJ	2013	SYNTAX
	Margolis K	2013	ALLHAT
	Menon V	2013	OAT
	Oparil S	2013	ALLHAT
	Piller L	2011	ALLHAT
	Poortvliet RK	2012	PROSPER
	Rahman M	2012	ALLHAT
	Robinson JG	2012	PHS
	Skolnick AH	2012	OAT
	Yamal J	2014	ALLHAT
Trial terminated early			
	Anderson GL	2012	WHI (Estrogen)
	Barsheshet A	2011	MADIT-II
	Goldenberg I	2010	MADIT-II
	Goodman PJ	2013	SELECT
	Molyneux A	2009	ISAT
	Molyneux A	2015	ISAT
	Nielsen PH	2010	DANAMI-2
	Rutqvist LE	2007	The Stockholm Breast Cancer Study
	Schierbeck L	2012	The Danish Osteoporosis Prevention Study
	Sever PS	2011	ASCOT-LLA
Active intervention in post-trial follow-up			
	Aroda VR	2015	DPPOS
	Connolly SJ	2013	RELY-ABLE
	Diabetes Prevention Program Research	2009	DPPOS
	Smith TR	2010	Combination therapy with amlodipine/valsartan in essential hypertension*

**open-label study investigating safety doses of intervention. Extension study of two previous RCT's. (Philipp T et al Clin Ther 2007; 29:563-80)*
