# Supplementary material for: Post-trial follow-up methodology in large randomised controlled trials: a systematic review
Source: Trials. 2018 May 30;19:298. doi: 10.1186/s13063-018-2653-0 (PMC5975470; doi:10.1186/s13063-018-2653-0)
Supplement: Supplementary file 4 — Risk of bias shown in each domain for an individual randomised controlled trial (RCT). Red indicates high risk, yellow indicates unsure and green indicates low risk. (PDF 173 kb) [file 13063_2018_2653_MOESM4_ESM.pdf]

Appendix C: Risk of bias shown in each domain for an individual RCT. Red indicates high risk, yellow indicates unsure and green indicates low risk.

Study	Allocation concealment?	Sequence Generation	Blinding of outcome assessors	Blinding of participants and personnel	Selective outcome reporting	Incomplete outcome data	Other sources of bias
Alan 2015	+	+	+	−	+	+	+
Arber 2011	?	+	+	+	+	+	−
Avenell 2012	+	+	+	+	+	+	+
BariInvestigators 2007	+	+	?	−	+	+	+
Breitner 2011	+	+	+	+	+	+	+
Carson 2015	+	+	+	+	+	+	+
Cauley 2013	+	+	+	+	+	+	+
Cherry 2014	+	+	+	+	+	+	+
Chew 2013	+	+	+	+	+	+	+
Chowdhury 2014	+	+	+	+	+	+	+
Cushman 2012	+	+	+	+	+	+	+
Dienstag 2011	+	+	+	?	+	+	+
DreamOnInvestigators 2011	+	+	+	+	+	+	+
Eastell 2015	+	+	+	+	+	+	+
Einstein 2011	+	+	+	+	+	−	+
Erdmann 2014	+	+	+	+	+	+	+
Ezzedine 2010	+	+	+	+	+	+	+
Ford 2007	+	+	+	+	+	+	+
Gada 2013	+	+	+	+	+	+	+
Gallagher 2014	+	+	+	+	+	+	?
Gluud 2008	+	+	+	+	+	+	+
Goldenberg 2009	+	+	+	+	+	+	+
Gordon 2012	+	+	+	+	+	+	+
Grau 2009	+	+	+	+	+	+	+
Grubb 2013	+	+	+	+	+	+	+
Hackshaw 2011	+	+	+	+	?	+	+
Halliday 2010	+	+	+	+	+	+	+
Hayashino 2009	+	+	+	+	+	+	+
Hayward 2015	+	+	?	?	+	+	+
HeartProtectionStudyCollaborative 2011	+	+	+	+	+	+	+
Henderson 2015	+	+	+	+	?	+	+
Hirsch 2007	+	+	+	+	?	+	+
Hochman 2011	+	+	+	+	+	+	+
Holman 2008	+	+	+	+	+	+	+
Hong 2014	+	+	?	+	+	+	+
Hornslien 2015	+	+	+	+	+	+	+
Johnson 2015	+	+	+	+	+	+	+
Jones 2015	+	+	+	+	+	+	+
Kostis 2011	+	+	+	+	+	+	+
Lai 2014	+	+	+	+	+	+	+
Laterre 2007	+	+	+	+	+	+	+
Leslie 2011	+	+	+	+	+	+	+
Leslie 2015	+	+	+	+	+	+	+
Lewis 2011	+	+	+	+	+	+	−
Lloyd 2013	+	+	+	+	+	+	+
Menne 2014	+	+	+	+	+	+	+
Milojevic 2016	+	+	+	+	+	+	+
Naunheim 2006	+	+	+	+	+	+	+
Ogihara 2011	+	+	+	+	+	+	+
Powell 2007	+	+	+	+	+	+	+
Radford 2014	+	+	+	+	+	+	+
Sedlis 2015	+	+	+	+	+	+	+
Tenkanen 2006	+	+	+	+	+	+	+
Wang 2015	+	+	?	+	−	+	−
Wanner 2005	+	+	+	+	?	+	+
Weston 2011	+	+	+	−	+	+	+
Whiteley 2014	+	+	+	+	+	+	+
Zoungas 2014	+	+	+	+	+	+	+
